# Exploring Proactive Behaviors of Employees in the Prevention of Burnout

**DOI:** 10.3390/ijerph16203849

**Published:** 2019-10-11

**Authors:** Madelon C.B. Otto, Nicole Hoefsmit, Joris van Ruysseveldt, Karen van Dam

**Affiliations:** Faculty of Psychology, Open University of the Netherlands, Valkenburgerweg 177, 6419 AT Heerlen, The Netherlands; nicole.hoefsmit@ou.nl (N.H.); joris.vanruysseveldt@ou.nl (J.v.R.); karen.vandam@ou.nl (K.v.D.)

**Keywords:** proactive behavior, burnout prevention, conservation of resources

## Abstract

Extensive evidence indicates that burnout can have detrimental consequences for individuals as well as organizations; therefore, there is a great need for burnout prevention. While burnout prevention interventions initiated by the employer have previously been studied, the proactive behaviors employees deploy themselves to prevent burnout have received less research attention. The purpose of this exploratory qualitative interview study was to enhance our understanding of the self-initiated actions employees undertake to prevent burnout, using the model of proactive motivation and conservation of resources theory as theoretical frameworks. Findings indicated that most participants reported to engage in specific kinds of proactive burnout prevention behaviors. The reported self-initiated proactive actions were aimed at maintaining and/or increasing resources and/or reducing demands in the work, home, and personal domain. The study contributes to the literature by linking the proactive motivation process to the prevention of burnout and by focusing on both work and non-work factors. Results of this study can be used in further research into the (effectiveness of) employees’ proactive burnout prevention behaviors and serve as a starting point for developing interventions aimed at enhancing proactive burnout prevention.

## 1. Introduction

Employee burnout is a topic of major interest and concern, given its high costs to both organizations and the individual employee [1,2,3]. The need to prevent burnout appears from numerous research findings showing that burnout has a negative impact on individuals’ physical health (e.g., prolonged fatigue, coronary heart disease, gastrointestinal issues) and psychological wellbeing (e.g., depression, insomnia), and is negatively associated with different work-related outcomes (e.g., lowered job performance, low organizational commitment) [1,2,4,5,6]. Burnout has recently been defined as a work-related condition consisting of exhaustion, loss of control over emotional and cognitive processes, and mental distancing [7]. These key symptoms may be complemented by secondary symptoms, such as feelings of depression, and behavioral and psychosomatic complaints of strain [8].

Ample evidence shows how a lack of resources to meet (high) job demands relate to employees’ burnout complaints [9,10,11]. While these studies thus indicate the actions organizations can take to prevent or reduce burnout risks, the actions that employees can take have received little attention [12]. This is surprising, since the individual outcomes of burnout can be so detrimental [6], that it is important to establish how employees themselves can intervene. Moreover, since personal and private factors have been found to influence the development of burnout [7,13,14,15], the initiatives of organizations alone may not be sufficient to prevent burnout. This is underscored by the fact that reviews of burnout prevention interventions conclude that employer-initiated programs have a lasting, but limited effect size [16,17]. Proactive actions of employees to prevent burnout may therefore complement organizations’ interventions [12].

Organizations increasingly expect anticipatory, self-initiated actions of employees to improve their situations [18]. Particularly since such proactive behaviors are considered to be crucial for organizations’ success, as they are seen as a driving force for innovation, adaptability and flexibility in the increasingly competitive and complex environments in which organizations operate nowadays [19]. Different proactive concepts (e.g., individual innovation, career initiative) have been developed and studied over the past years in different domains, showing that these behaviors can contribute to organizational and personal effectiveness [20,21]. Proactive concepts (such as job crafting and voice) have also been related to reduced levels of burnout complaints [22,23]. Although these concepts were not developed with the intention to prevent burnout, findings indicate that proactive behavior can be effective in the prevention of burnout.

The goal of this exploratory, qualitative study was to enhance our understanding of the self-initiated actions employees undertake to prevent burnout (henceforth referred to as ‘proactive burnout prevention’). The study contributes to the literature in three ways. First, the study combined principles of two theoretical frameworks to examine and understand employees’ proactive prevention behavior: Parker et al.’s [24] model of proactive motivation to investigate proactive behavior, and the conservation of resources (COR) theory [11,25] to examine the conservation of resources to prevent burnout. As far as we know, Parker et al.’s proactive motivation process [24] has not been empirically examined, nor has it been linked to the prevention of burnout. Second, this study applied an integrative approach to employees’ proactive burnout prevention behavior; in addition to factors within the work environment, this study’s focus was open to factors beyond the work situation that can additionally be used or changed by employees who proactively try to prevent burnout. Third, the findings of this study can serve as starting point to develop a tool to assess proactive burnout prevention, that can be used in future studies to further examine the effectiveness of this behavior. If proactive burnout prevention proves to be effective in preventing burnout, an intervention may be developed aimed at stimulating employees to deploy proactive behaviors that helps them to prevent burnout. 

### 1.1. Burnout Development and Prevention

According to COR theory, burnout is the consequence of a resource depletion process caused by prolonged exposure to stressors [1,11]. The basic principle of COR theory is that people are motivated to retain, protect, and build resources they value [11,25]. Resources refer to anything that is perceived by the individual to aid him or her to achieve his or her goals [26] and are therefore not limited to job resources, such as social support and control, but may also involve personal resources such as optimism and health [27,28,29]. Psychological stress occurs as a reaction to the environment in which there is a perceived threat to resources, an actual loss of resources, resources are inadequate to meet (high) demands (e.g., workload, pressure), or an investment in resources does not result in regaining resources [2,4,11]. Individuals strive to guard themselves from net resource loss and, when faced with a resource loss, react by trying to limit the loss and maximize the gain of resources, usually by deploying other resources [2,4]. If this investment in other resources does not result in the anticipated replenishment of resources, further resources may be depleted, and the continued loss may lead to downward loss cycles, ultimately, causing the employee to burn out [2,4,30]. 

Results of (meta-analytical) studies have shown the negative consequences of burnout for organizations and individuals [6,31], thereby underscoring the importance of burnout prevention. Consequences for the organization include increased absenteeism and higher turnover [1,9]. Individual outcomes involve a long list of possible physical consequences, such as: hypercholesterolemia, type 2 diabetes, hospitalization due to cardiovascular disorder, musculoskeletal pain, headaches, respiratory problems, severe injuries and mortality below the age of 45 years, and psychological effects, such as use of psychotropic and antidepressant medications, hospitalization for mental disorders and psychological ill-health symptoms [6]. In spite of these severe repercussions of burnout, review studies of burnout prevention interventions have found that only relatively few burnout preventions programs have been conducted, and even fewer have been evaluated [16,17,32]. Findings of these studies showed that burnout prevention interventions have small but lasting effects, yet more tailored strategies to prevent burnout are needed to improve the effectiveness of interventions [16,17].

Burnout prevention interventions have generally not included aspects beyond the workplace [16,17]. Although study findings have indicated that personal resources, home resources, and home demands can influence the development of burnout [33,34,35], these factors have received less research attention [36]. However, the effects of social and technological developments seem to make it increasingly important to include these factors when attempting to prevent stress and burnout [37,38]. For example, due to an aging population more employees have to combine their work with taking care of their elderly parents, which affects their wellbeing [37]. Also, electronics, such as smartphones and tablets, have enabled around-the-clock availability for work, making it more and more important to establish a good balance between one’s personal and professional lives to avoid resource depletion [38]. Including non-work factors in burnout prevention interventions may therefore enhance the effectiveness of these programs. Since these factors may be beyond the reach of the organization, this suggests that employee-initiated action may be essential for effective burnout prevention. 

### 1.2. Proactive Behavior and the Prevention of Burnout

Proactive behaviors have been described as anticipatory, self-initiated actions of employees aimed at changing or improving themselves and/or their environment [39]. Over the years, multiple types of proactive behaviors have been investigated and have been related to several positive work-related outcomes, such as job performance and innovation [40,41]. Proactive work behaviors have also been associated with lower levels of burnout [42,43], suggesting that employees themselves can take matters in their own hands to prevent burnout. It is however unclear what actions employees take to prevent burnout. 

In the context of burnout prevention, proactive behavior could focus on changing the employees themselves or their work or home environment to avoid resource depletion when dealing with high job demands [11]. Since proactive actions require resource consumption [40], they can be viewed as an investment in resources with the goal to maintain or gain resources [11]. In line with COR theory [11], this could be achieved by either undertaking actions aimed at a reduction in demands, thereby minimizing resource loss, or undertaking actions aimed at maintaining or increasing resources, thereby strengthening the resource pool. Since burnout develops gradually over time [11,44], this proactive conservation of resources should start in a timely manner, before resource loss leads to prolonged periods of stress, which may offset further cycles of resource loss and eventually leave the employee burned out [4,11,25]. The present study, therefore, focused on the stage when employees feel threatened by resource loss (expressed by a higher perceived level of exhaustion), and there is still opportunity for initiatives to prevent resource depletion and burnout [26,45].

According to Grant and Ashford [46], proactivity is not a particular set of behaviors, but more usefully can be considered as a process involving anticipating, planning and striving to have an effect. Following, Parker et al. [24] have described a model of proactive motivation involving two elements: goal setting and goal striving. The goal-generation stage consists of anticipating desired current or future states or outcomes and developing strategies to achieve those goals. The goal-generation stage holds two processes: envisioning and planning [46,47]. Envisioning refers to perceiving and identifying a current or future problem and picturing a different future that can be brought about by actively addressing the problem [24,48,49]. Planning means that the individual decides which actions must be taken to change the self and/or the situation in order to achieve the envisioned future [24,48]. The second stage, goal-striving, involves the actual behaviors and the monitoring of these behaviors to attain the set goals [24]. The goal-striving stage also includes two processes: enacting and reflecting [47]. Enacting concerns the overt actions people take to change themselves and/or their situation to reach the proactive goals they have set [24,48]. Reflecting involves the efforts of an individual to understand successes, failures and consequences of one’s proactive behaviors [24,48]. 

**Figure 1:**
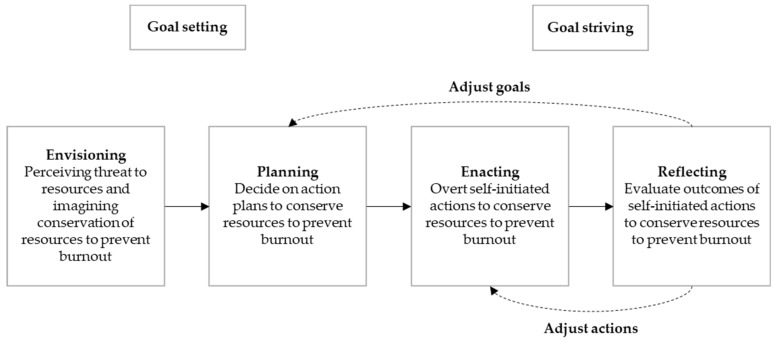
Model of proactive burnout prevention process (adapted from Parker et al., 2010).

The current study applied the goal setting and goal striving principles of this model of proactive motivation [24] to the conservation of resources to prevent burnout [11] and used the adapted model (see Figure 1) as theoretical approach to explore employees’ proactive burnout prevention behaviors. In this context, envisioning referred to experiencing feelings of distress and identifying the need to prevent burnout, planning involved determining proactive action plans to conserve resources to prevent burnout, enacting consisted of the actual proactive actions plans pursued to conserve resources to prevent burnout, and reflecting included reviewing outcomes of the proactive behavior to prevent burnout and establish whether actions need to be continued or modified. The reported proactive actions were categorized to enable further investigation of proactive burnout prevention.

## 2. Materials and Methods

### 2.1. Design and Participants

This study used a qualitative research design for gaining in-depth knowledge on the employees’ perspective [50,51]. Semi-structured interviews were conducted between February and May 2018. To ensure complete and transparent reporting, the consolidated criteria for reporting qualitative research (COREQ) checklist was used as a guideline to perform this study [52]. The study was conducted in accordance with the Declaration of Helsinki [53], and the protocol was approved by the Ethical Committee of the Open University of the Netherlands (correspondence 21 February 2018, registration number: U2018/01317/HVM). All participants signed an informed consent for inclusion prior to the start of the interview.

Participants met the following inclusion criteria: employees (>18 years), who regularly experience feelings of exhaustion at the end of their working day (in line with item 3 of the Dutch version of the need for recovery scale of the questionnaire of experience and evaluation of work; QEEW: ‘Because of my job, at the end of the working day I feel rather exhausted’ [54]). Participants did not meet the exclusion criteria: employees who have experienced a long period (>6 weeks) and complete absence of work due to burnout or are on sick leave at the time of the study. Purposive sampling was used to obtain the perspectives of employees with varying jobs, gender, ages and industries to increase transferability. 

The participants were recruited with the help of several occupational health professionals and counsellors working at different occupational health service organizations and from the personal network of the participants. The occupational health professionals and confidential counsellors were asked to identify potential participants who met the inclusion criteria and did not meet the exclusion criteria, and to inform them verbally of the study and by providing them with an information letter that was composed by the researchers for this purpose. This letter provided these potential participants with detailed information about the study and emphasized that participation was voluntary, confidential and could be stopped at any time. Those who were willing to take part in the study, were asked to contact the researchers themselves by e-mail. 

Data collection ended after 12 interviews, as saturation had been reached (no new information emerged from the last two interviews) [55]. The sample consisted of 6 men, and 6 women, with ages ranging from 23 to 61 years old (M = 48.5; SD = 12.1). Education level varied from lower professional education (N = 5) to higher professional education (N = 4) and university (N = 3). Eight participants worked fulltime, four worked part-time. Participants came from six different industries (healthcare, education, logistics, information & communication, business services and charity).

### 2.2. Data Collection

The interviews lasted approximately one hour and were held at locations of participants’ choice (e.g., at their workplaces or homes). All interviews were conducted by one researcher who had undergone formal interview training and had prior experience in conducting interviews. Before starting the interviews, participants were informed of the objective of the study and were given some background information on the education and working experience of the interviewer. Nine of the 12 interviews were voice-recorded. One participant did not consent to the voice-recording due to privacy concerns, and two interviews were not voice recorded due to a failure (in the use) of the voice-recording device. During these three interviews, notes were made, and summaries were sent to the participants for comments and corrections. Additional field notes were made by the researcher during and after the interviews about the conduct and non-verbal behavior of the participants. 

The use of a topic list ensured that all relevant issues with regard to the research questions were discussed. Some example topics were: ‘Description of the stressful situation’, ‘Description of what helps the employee to prevent burnout’, and ‘Description of actions the employee may take or may have taken to conserve resources’. Employees were asked to talk about their experience with high stress situations, and how they actively manage their demands and resources to prevent burnout. All topics were explained in plain language. Questions were posed in a neutral and open-ended manner, and suggestive questions were avoided. Participants were asked questions such as: Which …? How …? Can you give an example? Is there anything else …? to make sure the information given was complete and accurate. 

A concept topic list was pilot tested to ensure that the gathered data would be appropriate to answer the research questions. For this pilot test two employees (recruited within the personal network of the interviewer), who had experienced periods of high stress and burnout, were interviewed. The transcripts of these interviews were thoroughly examined by the researchers and resulted in an optimization of the inclusion and exclusion criteria, the topic list, and the interview style, as previously described. The data obtained in the pilot test were not included in the data analysis because of the adjustments to the inclusion and exclusion criteria. 

### 2.3. Data Analysis

The interviews were voice-recorded and transcribed verbatim. The qualitative data analysis software MAXQDA 2018 [56] was used to code meaningful fragments in the text, conform the thematic analysis method described by Braun and Clarke [57]. This method was chosen because it facilitates a theoretically flexible approach to the coding of data [57]. Applying this method to the current study implied that the researchers interpreted the data with theoretical sensitivity to the model of proactive motivation [24] and COR theory [11]. Specifically, it was noticed whether participants reported self-initiated actions to prevent burnout, and if their conduct reflected the underlying systems and elements of the model of proactive motivation as described by Parker et al. [24]. 

The analysis process involved six phases: (1) familiarization with the data, (2) generating initial codes, (3) searching for themes, (4) reviewing themes, (5) defining and naming themes, and (6) producing the report [57]. Phase (1): The transcripts and summaries were copied into a MAXQDA datafile and read and reread multiple times for familiarization with the data. Phase (2): In this initial coding phase, all relevant fragments containing information on (the process of activating) self-initiated actions and behaviors to conserve resources to prevent burnout were given a label. Phase (3): In this phase, the different codes were abstracted, defined and categorized into potential main and sub-themes using the elements of the goal setting (envisioning, planning) or goal striving (enacting, reflecting) stages of the proactive process [24]. This included identifying resources and demands that were targeted to prevent burnout [11]. Phase (4): This phase involved reviewing the coded data extracts of each theme and rereading the data set to get a clear overview of the different themes and how they may fit together in relation to the research question. This resulted in combining, removing, and separating themes. Phase (5): In phase five, the themes were further defined, refined and named. Main categories were identified, consisting of a varying number of self-initiated actions as sub-themes. Phase (6): To provide evidence of the existence of each theme within the different categories, statements from the raw data were extracted. These statements were included in the reporting of the results to substantiate the findings in relation to the research question.

Throughout the process, the researchers remained sensitive to data that did not fit within the framework of Parker et al. [24] or with COR theory [11] but still might be relevant for the research purpose. Collection and analysis of data was alternated in what Braun and Clarke [57] describe as a ‘recursive process’, during which the researchers continuously and repeatedly reflected on, compared, discussed and adjusted the coding (to ensure confirmability) until consensus on the outcomes was reached.

## 3. Results

The results are presented according to the four stages of Parker et al.’s [24] proactive process model: envisioning, planning, enacting, and reflecting (see Figure 1).

### 3.1. Envisioning

The data were analyzed to establish whether the participants identified the need for proactive burnout prevention. All participants reported experiencing feelings of distress and unrest due to (sustained) high demands: *“… my head is full, I am tired, not sleeping well. …lying awake at night thinking about everything.”* (Participant8). Three quarters of the participants stated that these demands were not only related to their job (e.g., high workload): *“… I just have too much work to do. I cannot finish it all within the agreed timeframe …”* (Participant12), but also involved requirements at home (e.g., having to take care of a sick or disabled family member): *“… our parents are still alive … they are in their nineties and require care …”* (Participant10), and/or distress induced by a stressful event (e.g., divorce): “*Three children to raise on your own …*” (Participant1). 

However, findings indicated that not all participants identified the need to proactively intervene to prevent burnout as response to these high demands. As a result, two groups could be distinguished. The first group, henceforth referred to as ‘interventionists’, consisted of two thirds of the participants, who reported to realize that they had to take action to prevent burnout: *“When you experience stress and you do not change anything to the situation, or yourself, then it is inevitable that the camel’s back will break.”* (Participant1). Most of the interventionists stated that while envisioning they expected advantages of taking action to prevent burnout, such as increased support: *“So I may resort to somebody that will fulfill an ‘assistant-to’ role … I think that may help.”* (Participant2). The frequency with which these participants reported to envision, varied from doing it regularly: *“At the end of each week I take a moment to self-reflect …”* (Participant5), to only doing it when experiencing increased feelings of distress or fatigue: *“… a kind of a nervous feeling … I take notice of it and perceive it as a signal to reflect … ”*(Participant2).

The second group, henceforth referred to as ‘non-interventionists’, consisted of the remaining one third of the participants, who had a different stance. Although they also mentioned to perceive problems due to (prolonged) high demands and seemed aware of the concept of burnout, they were not contemplating the prevention of a possible future burnout: *“Yes, I am aware, but it is more a kind of theoretical awareness.”* (Participant6). Half of the non-interventionists reported the need to gain favorable judgements of their competence: *“Apparently I like to score, so I can show that I am contributing and that my contribution is appreciated. To show I am relevant …”* (Participant9), the other half of this group declared to be somewhat passive and defensive when asked how they dealt with high job demands: *“… I often just ignore it … I don’t feel like dealing with it.”* (Participant3). As a result, the non-interventionists did not set proactive goals to prevent burnout and are therefore not included in the following description of the outcomes regarding the planning, enacting and reflecting stages. The actions and behaviors that the non-interventionists used for reactive coping with their high (job) demands will be described at the end of the results section. 

### 3.2. Planning

The data were analyzed to determine the proactive action plans the interventionists worked out to prevent burnout. The results showed that although the interventionists consciously made decisions which action plans to pursue to avoid resource depletion, they did not explicitly report on their thought process of how these decisions came about. The following six action plans could be distinguished: (1) Maintain/increase job resources. The interventionists reported the need to maintain/increase their resources at work, for instance, the need to maintain/increase job control*: “I created my own document in Excel… to get a better overview of my tasks, so I feel more in control.”* (Participant5). (2) Maintain/increase job challenges. The interventionists mentioned to consciously look for or perform tasks that boost their energy level: *“I have asked my boss to assign me extra tasks that energize me”* (Participant4). (3) Reduce job demands. This included reducing the actual amount of work that had to be done: *“This and this and this is what I am going to do, and the rest is less important, so I will not do …”* (Participant1). (4) Maintain/increase home resources. This included actions to maintain/increase home autonomy: *“For instance, having to eat at the in-laws every Monday. I do not want that.”* (Participant7). (5) Reduce home demands. This referred to switching off from work and/or reducing duties and requirements at home to be able to deal with high job demands: *“Well, the laundry needs to be done, dinner needs to be cooked. That is the reason that I completely outsourced that. Otherwise it is impossible.”* (Participant2). (6) Maintain/increase personal resources. This referred to actions to stay physically as well as mentally fit for the job: *“I make sure that I get enough sleep, to stay physically and mentally healthy...”* (Participant5).

### 3.3. Enacting

All interventionists reported taking action to prevent burnout not only in the workplace, but also in the home and personal domain. The reported proactive actions the interventionists claimed to take or have taken to prevent burnout are depicted in Table 1 and will be outlined below. Table 1 also shows how many and which participants engaged in each identified proactive action. 

#### 3.3.1. Maintain/Increase Job Resources 

All interventionists reported taking self-initiated actions to maintain/increase various job resources to prevent burnout. For example, maintain/increase job control*: “I have started to schedule my tasks more conscientiously to feel more in control.”* (Participant12). Also, maintain or increase social job resources, such as asking co-workers for support: *“I also often exchange thoughts with co-workers who perform a similar role, because they face the same issues.”* (Participant8) or approach the supervisor for support: *“If I feel that I cannot finish my work on time, I ask my supervisor for help.”* (Participant12). Seeking feedback was another job resource that was mentioned by the interventionists that helped them to deal with high pressure: *“It helps to spar with co-workers…I had this situation, and this is what I did about it. What do you think?”* (Participant2).

#### 3.3.2. Maintain/Increase Job Challenges

Almost half of the interventionists mentioned to actively engage in or ask for tasks that energize them to prevent burnout: *“I have become more selective in the assignments that I accept. … So, I take on assignments I like for clients I like and that energizes me.”* (Participant10).

#### 3.3.3. Reduce Job Demands

Various actions were reported by all the interventionists to reduce their job demands in an attempt to avoid burnout. For instance, setting priorities: *“I also make choices and set priorities…, otherwise it is too much.”* (Participant2); delegating or rejecting tasks: *“I have withdrawn myself from the branch committee because I do not have the time.”* (Participant11); or moving tasks forward in time to control their workload: *“The peace I feel that when I cannot make it in time, I call and say, hey, it is going to be finished a bit later.”* (Participant10). 

#### 3.3.4. Maintain/Increase Home Resources 

All of the interventionists stated undertaking actions aimed at maintaining/increasing home resources to prevent burnout. They reported doing this by maintain/increasing home autonomy: *“I don’t make pre-arrangements for Tuesday nights. If I feel like it, I call friends to set something up, if I am too tired, I do nothing”* (Participant5); engaging in relaxing activities: *“I consciously go home at lunchtime to walk my dog. This relaxes me.”* (Participant4); and maintaining/increasing social support from family/friends: *“If I experience stress signals, I talk about it with my wife and this helps me.”* (Participant12).

#### 3.3.5. Reduce Home Demands

All but one of the interventionists declared taking proactive actions aimed at maintaining or reducing home demands in order to prevent burnout. For example, by managing duties at homes: *“Our parents are in their nineties, so that requires some family care… but I also want my own time. So, I do not want to have three nights of social activities… So, we regularly discuss the agenda.”* (Participant10), and/or make time to switch off from work both figuratively: *“You really need to switch off, to make sure you create some space to be able to go on.”* (Participant2), as well as literally: *“I have a separate telephone for work that I switch off when I get home.”* (Participant4).

#### 3.3.6. Maintain/Increase Personal Resources 

All interventionists reported to take proactive action to maintain or improve their physical health, as important personal resources to avoid resource depletion: *“I believe it is very important to at least stay physically healthy. That gets me through this period.”* (Participant11). In order to stay physically healthy interventionists mentioned engaging in some form of physical activity that helps them to contain their energy level and stay fit: *“For me, an important way to deal with that is exercising… That gives me energy.”* (Participant10). In addition, they claimed to have consciously adopted a healthy lifestyle and to maintain this as resource to maintain their health and improve their energy level: *“I try to eat healthy, because I notice that I get a lot more energy out of that, than when I heat up six frozen pizza’s weekly.”* (Participant7). 

All but one of the interventionists declared to undertake proactive actions to maintain or improve their mental health to prevent burnout. These proactive actions consisted of cognitive reappraising their stress provoking situations or thoughts: *“Clear your head, suddenly envision other possible solutions, and make things less heavy.”* (Participant10); consulting a coach to help them put things into perspective: *“Because you can develop a sort of tunnel vision when the pressure remains high. If you cannot zoom out now and then, it is better to talk to someone and put things into perspective.”* (Participant2); creating peace of mind, for instance by actively searching for and imagining alternative job opportunities or sources of income: *“If the workload becomes too high, I have in the back of my head that I can always start working for myself.”* (Participant4); proactively engaging in some form of mindfulness activity to prevent burnout: *“We also do breathing exercises that I sometimes also do during the day. Then I check, hey, how is my breathing? Is it too shallow? Maybe I need to do something with it, that I am being too restless.”* (Participant7); and stimulating a positive mindset: *“There is a solution for every problem.”* (Participant1). 

### 3.4. Reflecting

The data were analyzed to establish whether the interventionists experienced their actions to be successful in that they indeed were able to avoid resource depletion to prevent burnout, or that they needed to modify the proactive goals, or the efforts to achieve those goals. All interventionists claimed that most of their actions were successful in achieving their proactive goals. For instance, engaging in physical activity, thereby successfully conserving personal resources (i.e. physical and mental health) to deal with job demands: *“… when you have exercised, you think, yes, this is what I needed. I have taken time for myself and now I can deal with the mailbox. And that is helpful.”* (Participant7). Nonetheless, half of the interventionists mentioned actions that were unsuccessful in achieving their proactive action plans. These actions were all related to asking the supervisor for social support to get the work done: *“… we indicated, it doesn’t work, we need help … “*(Participant1). Two different responses to these unsuccessful actions could be distinguished: either the participants mentioned to replace the action with another action aimed at the same proactive action plan, or the participants stated to modify their proactive action plan, and subsequently adjusted efforts to achieve that goal (see dotted arrows from reflecting to enacting and from reflecting to planning respectively in Figure 1). An example of the first response was, instead of maintaining/increasing social support by asking their supervisor for help, achieve this by seeking support from co-workers: *“When there are moments that we proclaim: we need support, we need help … that it is often the case that this support is just not given. … So, we solve it together. Because it is a pleasant tight knit team it works.”* (Participant1). An example of the second response was, changing the proactive action plan from (unsuccessfully) maintaining/ increasing social job resources: *“I have told my boss, that this causes me a lot of distress…, but she is not going to say, I will handle it, leave it to me.”* (Participant11), to maintaining/increasing work resources by for instance rejecting tasks to maintain/increase control over the amount of work to be done: *“This was a huge project, that would take up a lot of my time. So, I told my boss, sorry, but I let this one pass. She wanted me to participate, but I told her it is going to take up way too much of my time. It’s impossible.”* (Participant11).

### 3.5. Actions and Behaviors of ‘Non-Interventionists’

Four participants did not mention a clear motivation to prevent burnout and did not envision conserving or gaining resources. These non-interventionists seemed to use reactive coping strategies. Their behavior appeared to be aimed at alleviating stress symptoms, instead of proactively preventing them: *“Most of the time I come home and think, choke on it, I don’t feel like doing anything anymore. I am going to sit on the couch, turn on the tv and that’s it.”* (Participant8). Although some of the reported actions of the non-interventionists seemed similar to the actions stated by the proactive participants, the actions of the non-interventionists were all aimed at symptom relief, and not at changing themselves or their environment to solve or avoid problems in order to prevent burnout. 

All of the non-interventionists stated to undertake some kind of activity with friends or family to relax after a stressful day at work: “*To relax, … visit friends and watch football together.”* (Participant8). Three quarters of the non-interventionists reported drinking alcohol as a means of coping with work stress: *“I actually want to say something really bad right now. I notice that on Friday night, when I have had to deal with lot of stress during the week and I drink some wine, that helps.”* (Participant9). In addition, half of this group declared to exercise to release tension: *“Exercising… that helps me by being able to relax for a moment.”* (Participant6). Also, half of the non-interventionists mentioned to engage in some kind of mindfulness related activity in an attempt to relieve feelings of discomfort: *“I know that when I start to feel uncomfortable, that I can, by doing certain breathing exercises and relaxing certain parts, I can deal with it.”* (Participant3). Lastly, half of this group of participants reported to pursue their hobbies to cope with feelings of distress: *“What also often helps, I like to knit and crochet and such. … A kind of yoga for the head.”* (Participant3).

## 4. Discussion

The aim of this study was to explore employees’ proactive burnout prevention behaviors. Semi-structured interviews were conducted with 12 employees who reported to feel regularly exhausted at the end of a working day but had never experienced burnout.

Findings of this study showed that two-thirds of the participants (referred to as ‘the interventionists’) stated to deliberately undertake self-initiated actions with the aim to maintain or increase their resources and/or reduce their demands in order to avoid resource depletion. This result is in line with Parker et al.’s [24] notion that job stressors can prompt proactive behavior, to decrease the discrepancy between a current and desired situation. Outcomes of this study further indicated that ‘how’ the interventionists engaged in these proactive behaviors could be described along the four stages of the goal-driven proactive process modelled by Parker et al. [24]: envisioning, planning, enacting and reflecting (see Figure 1). 

Envisioning entailed in this case that the interventionists reported to feel a threat to their resources due to high demands and realized the necessity and possibility to take action to conserve resources in order to prevent burnout. In the planning process, the interventionists indicated to decide on the following action plans to avoid resource depletion: maintain/increase job resources (e.g., job control), maintain/increase job challenges (e.g., energizing tasks), reduce job demands (e.g., workload), maintain/increase home resources (e.g., home autonomy), reduce home demands (e.g., household chores), maintain/increase personal resources (e.g., physical health). The enacting stage consisted of the overt actions the interventionists reported to take to achieve the action plans identified in the goalsetting process. The reported proactive actions were categorized into demands and resources targeted actions in the work, home and personal domain (see Table 1). For instance, delegating or rejecting tasks with the aim to reduce job demands, and/or consciously plan leisure activities to maintain home resources, and/or do physical activity to increase personal resources to remain physical healthy and energized to take on demands. Finally, in the reflecting stage the interventionists mentioned to review their actions in terms of experienced success or failure to achieve the aforementioned action plans in order to determine whether goals or actions needed to be modified. 

The findings of this study suggested that the feelings of exhaustion that the participants reported, did not only stem from their job demands, but in almost all cases arose from a combination of high demands in the workplace and requirements at home (e.g., having to take care of a sick or disabled family member, facing a stressful event). Consequently, the planned actions indicated that the interventionists not only targeted job resources, but also personal and home resources in their attempt to avoid resources depletion. These results suggest that in order to prevent burnout, it may be important to take an integrative approach and not only focus on factors within the work environment, but also include factors beyond the work situation [13,14]. To further reinforce this point, the findings indicated that employees simultaneously undertake proactive actions to avoid burnout in several domains, thereby suggesting that employer-initiated actions in the workplace alone may not be sufficient to effectively prevent burnout.

All resources that the interventionists reported to proactively target to prevent burnout have in previous research been linked to reduced levels of burnout. Ample research has established the importance of sufficient job resources to guard against high job demands [9,10]. Research in the field of work-home interference has shown that home resources, such as social support from family and friends are an important protective factor to prevent burnout [13,58]. Several personal resources, such as physical health and psychological wellbeing have been negatively associated with burnout [2,59]. In addition, higher job and home demands have been linked to higher levels of burnout [15,33], indicating that proactively reducing these demands may indeed result in diminished burnout complaints. Although previous research already found relationships between work resources, social job resources, personal resources, home resources and burnout, it has not been investigated before, whether and how employees proactively conserve these resources to prevent burnout. The findings of this study thus contribute to scientific knowledge by linking the proactive motivation process [24] to the prevention of burnout and by focusing on work as well as non-work factors that can be used or changed by employees to proactively try to prevent burnout.

The participants who did not indicate to engage in proactive burnout prevention (referred to as the ‘non-interventionists’), seemed to use reactive copings strategies, such as mental disengagement and/or alcohol disengagement (e.g., practicing hobbies, drinking alcohol) [60] to deal with their high demands. The actions and behaviors of this group seemed more focused on reactively attempting to reduce or eliminate physical and psychological strain symptoms, than on proactively addressing the causes of these high demands to prevent burnout. To be able to sustain their ability to work, the non-interventionists appeared to attempt to replenish and restore depleted resources on a daily basis. It is questionable whether this approach helps to prevent burnout in the long term. Previous research has shown that individuals who have to deal with high job demands, may not recover sufficiently from work and experience increased levels of burnout [61]. In case of prolonged periods of high demands, it may therefore be more successful to proactively solve the underlying problem, than to repeatedly try to relief the (strain) symptoms.

The proactive behaviors to prevent burnout reported in this study, show resemblance to Tims and Bakker’s [62] description of the proactive behaviors of job crafting based on the JD-R model. In fact, proactive burnout prevention seems to capture the conceptualization of job crafting by Tims and Bakker [62] in that it also involves increasing job resources, increasing job challenges, decreasing job demands, and both consider employees’ wellbeing as the outcome of proactive behavior. However, unlike job crafting, the reported proactive burnout prevention behaviors do not only focus on work-related factors but include personal factors and aspects outside the workplace as well. Another difference between the two concepts is that the goal of job crafting as stated by Tims and Bakker [62] is to achieve a better fit between the job and the employees’ personal knowledge, skills or interests, and not to prevent burnout. In addition, envisioning seems to be a prerequisite for deploying proactive burnout prevention, which seems less (explicitly) the case with regard to job crafting. So, although elements of proactive burnout prevention may show similarities to aspects of job crafting, the differences in aim and scope appear considerable and seem to justify it being considered as a separate type of proactive behavior. 

### 4.1. Limitations

This study has some methodological limitations. Findings of this study indicated the different kinds of proactive behaviors employees engage in to prevent burnout. Although the participants claimed that these behaviors successfully helped them to prevent burnout, we cannot draw conclusions about the effectiveness of proactive burnout prevention based on our research design. This requires a longitudinal quantitative design that includes participants from different stages of burnout.

Moreover, the sample size of this study was limited to 12 employees. Yet, saturation was reached in the eighth interview. This is in line with the findings of a review study by Guest et al. [55] into saturation and variability. They found that saturation can occur within 12 interviews and elements for key themes can already be presented after six interviews. Data collection and analyses were alternated to ensure that saturation could properly be established. Table 1 shows that all proactive actions were identified by at least three participants, and all participants reported multiple proactive actions. 

In addition, the research sample did not contain any blue-collar workers, which limits the transferability of the study findings to this population. To illustrate, the interviewed white-collar workers did not report any physical job demands that blue-collar workers are more likely to face. This may have resulted in additional or different actions they might deploy to conserve physical resources to prevent burnout. Proactive behaviors also require some degree of autonomy which might be lacking in blue-collar jobs [63]. 

### 4.2. Directions for Further Research

This explorative study can serve as a starting point for investigating proactive burnout prevention. Multiple directions for further research can be identified. A measure that assesses employees’ proactive burnout prevention behaviors should be developed and validated which will enable the examination of the effectiveness of these behaviors. If proactive burnout prevention is indeed found to be effective in preventing burnout, an intervention can be developed and implemented to enhance these behaviors. Yet, this does not imply that proactive burnout prevention is the responsibility of the employee only. As stated, proactive behavior can be influenced by individual differences, as well as contextual factors, and it is therefore important that a work environment is created by the employer in which employees feel encouraged to be proactive [64].

Most participants in this study indicated to engage in proactive burnout prevention behaviors. Some other participants seemed to resort to reactive coping as response to high demands at work and requirements at home. More research is needed to describe and understand the differences between these two groups. What contextual and individual factors influence employees’ motivation (or lack thereof) to engage in proactive burnout prevention?

Further research is also needed to determine how proactive burnout prevention relates to other types of proactive behavior. As discussed, proactive burnout prevention shares some similarities with job crafting [62], and previous research has found proactive work behavior, such as job crafting and voice to be associated with lower levels of burnout [22,42].

## 5. Conclusions

Findings of this exploratory qualitative study showed that the interviewed employees engage in specific kinds of proactive behaviors to prevent burnout. Participants reported that not only work factors, but also private factors contributed to feelings of exhaustion, implying that employer-initiated actions in the workplace alone may not be sufficient to prevent burnout, and employees themselves may need to intervene as well. Results of this study indicated that employees indeed undertake proactive actions aimed at maintaining and/or increasing resources and/or reducing demands in the work, home, and personal domain to prevent burnout. Moreover, participants stated to simultaneously undertake actions in more than one domain. Findings of this study can be used in further research into the (effectiveness of) proactive burnout prevention behaviors. If research demonstrates that specific burnout prevention behaviors can be effective in preventing burnout, this may inspire the development of an intervention that promotes these self-initiated behaviors.

## Figures and Tables

**Table 1:** Proactive burnout prevention planning and enacting stages and the participants identifying each proactive action.

Domain	Planning (Action Plans to Prevent Burnout)	Enacting (Overt Actions/Behavior to Prevent Burnout)	Participants
Work	Maintain/increase job resources	Increase/maintain job control	All ^1^
		Increase/maintain supervisor social support	1, 4, 5, 7, 11, 12
		Increase/maintain coworker social support Seek feedback	1, 2, 4, 5, 7, 11, 12 1, 2, 8
	Maintain/increase job challenges	Seek/perform tasks that energize	2, 5, 10
	Reduce job demands	Reduce job demands	All ^1^
Home	Maintain/increase home resources	Maintain/increase home autonomy Engage in relaxing activities Maintain/increase family/friends social support	1, 2, 4, 5, 7, 10 2, 4, 11, 12 All ^1^
	Reduce home demands	Reduce home demands	1, 2, 4, 5, 7, 10, 11
Person	Maintain/increase personal resources	Maintain/improve physical health Maintain/improve mental health	All ^1^ 1, 2, 4, 5, 7, 10, 11

^1^ Note. ‘All’ refers to all interventionists, who indicated to take proactive action to prevent burnout, as opposed to the non-interventionist, who did not indicate to take proactive actions to prevent burnout.
